# Impact of right ventricular dysfunction assessed by cardiac magnetic resonance imaging on prediction of short term and long term prognoses after acute inferior myocardial infarction

**DOI:** 10.1186/1532-429X-13-S1-P182

**Published:** 2011-02-02

**Authors:** Takayuki Onishi, Tomoyuki Kawashima, Hirotaka Muramoto, Hiroaki Nakamura, Yasutoshi Nagata, Isshi Kobayashi, Yuko Onishi, Shigeo Umezawa, Akihiro Niwa

**Affiliations:** 1 Hiratsuka Kyosai Hospital, Hiratsuka, Japan

## Objective

We assessed the prognostic importance of right ventricular (RV) involvement in patients with acute inferior myocardial infarction (MI).

## Background

RV infarction contributes markedly to hemodynamic instability, atrioventricular conduction block and in-hospital mortality in patients with inferior MI. Cardiac magnetic resonance imaging (MRI) is the superior imaging technique for assessing RV involvement because of its high spatial resolution.

## Method

We analyzed 20 consecutive patients (14 male; age 64 ± 12 years) with first acute inferior MI due to proximal right coronary lesion. All patients were assessed with cardiac MRI after primary percutaneous coronary intervention and followed up for a median (25^th^, 75^th^ percentiles) of 6 (5, 6) months. We assessed the association of RV function with length of cardiac care unit (CCU) treatment as an index of short-term prognosis by using multiple regression analysis, and used Kaplan-Meier analysis to demonstrate the association of RV dysfunction with occurrence of heart failure (HF) after discharge as an index of long-term prognosis.

## Results

RV involvement was diagnosed with delayed enhancement MRI in 11 patients (55%). Patients with RV involvement had lower RV ejection fraction (EF) (32.4 ± 8.6 % vs 43.2 ± 7.7 %; p = 0.009) and longer duration of CCU treatment (5.3 ± 2.3 days vs 3.5 ± 1.9 days; p = 0.038) than those without RV involvement, although there were no significant differences regarding clinical, angiographic and the other MRI characteristics such as left ventricular parameters. By multiple regression analysis, RVEF was an independent predictor of length of CCU treatment (standardized partial regression coefficient -0.799, partial regression coefficient -0.127; 95% confidence interval -0.222 to -0.031; p = 0.017). Kaplan-Meier analysis demonstrated that RVEF <30% was associated with increased occurrence of HF in chronic phase (p = 0.003).

## Conclusion

RVEF in patients with acute reperfused inferior MI is an important predictor of short-term prognosis and patients with lower RVEF had more occurrence of HF at long-term follow-up than those without lower RVEF. Evaluation of RV with cardiac MRI can improve risk stratification and patient management after acute inferior MI.

